# Phytochemicals and syneresis of osmo‐dried mulberry incorporated yoghurt

**DOI:** 10.1002/fsn3.645

**Published:** 2018-04-16

**Authors:** Aliza Sigdel, Pravin Ojha, Tika B. Karki

**Affiliations:** ^1^ Department of Food Technology National College of Food Science and Technology Tribhuvan University Kathmandu Nepal; ^2^ Food Research Division Nepal Agricultural Research Council Lalitpur Nepal; ^3^ Departmet of Biotechnology Kathmandu University Dhulikhel Nepal

**Keywords:** anthocyanin, antioxidant activity, ascorbic acid, lactic acid bacteria count, total phenol

## Abstract

The research was aimed to study the effect of the addition of Osmo‐air‐dried mulberry (TSS 29.33%) in yoghurt on syneresis and a bioactive component of yoghurt. Two types of yoghurts, with or without Osmo‐dried mulberry, were developed using standard culture (*Streptococcus thermophilus* and *Lactobacillus bulgaricus*), and changes at refrigerated temperature (<5°C) were studied. Fruit yoghurt showed high total soluble solids (TSSs) and low‐fat content (dry basis) (17.67% and 11.84%) compared with normal yoghurt (9.5% and 17.21%). The addition of fruits increased the ascorbic acid (0.77 to 5.96 mg/100 g yoghurt), anthocyanins content (0 to 7.9 mg/100 g yoghurt), total phenol content (TPC) (6.63 to 68.03 mg GAE/100 g yoghurt), and antioxidant activity (20.73% to 47.6% radical scavenging activity) in yoghurt. During 18^ ^days of storage at refrigerated condition (<5°C), the acidity of all samples increased, while pH decreased. Syneresis increased with a storage period in control samples while fruit incorporated yoghurt showed decreased syneresis with time. The viability of lactic acid bacteria (LAB) count went on decreasing at similar rates for both with and without the Osmo‐dried mulberry incorporated yoghurt. There is an ample opportunity for utilization of Osmo‐air‐dried mulberry in yoghurt to prevent syneresis during storage with increased bioactive components.

## 1 INTRODUCTION

Yoghurt is a dairy‐based product, which popularity is increasing day by day due to its nutritional and therapeutic characteristics (Nazni & Komathi, 2014; Yousef, Nateghi, & Azadi, 2013). Yoghurt is a lactic acid fermented product from milk with an acidic taste (Aswal, Anubha, & Priyadarshi, 2012; Hassan & Amjad, 2010). Fruit and fruit preparation is used in yoghurt not only to improve the nutritional properties, but these fruit yoghurts also possess phytochemicals such as vitamin C, carotenoids, phenolic components, and antioxidant component (Ariaii, Mahmoudi, & Amoli, 2011; Bae & Suh, 2007; Nazni & Komathi, 2014; Shori & Baba, 2014). Mulberry belonging to family Moraceae and Genus Morus is grown in Nepal at an altitude of 500–2,000 m from tropical to subtropical climates (Shrestha, 2006). Mulberry is a seasonal fruit and is extremely perishable with limited market demand, so the postharvest loss is high (Doymaz, 2004). Its utilization is limited to silkworm and feed (Mehla, Patel, & Tripathi, 1987).

It is rich in anthocyanin and other bioactive components, which may provide beneficial health‐promoting properties (Kako, 2012; Kao, 2003). Berries possess a significant amount of anthocyanin, which possesses antioxidant properties (Brito, Areche, Sepúlveda, Kennelly, & Simirgiotis, 2014; Bunea et al., 2013). Osmo‐air drying not only results in better nutrient retention but also maintains the good integrity of the fruit and has good rehydration property (Djendoubi, Boudhrioua, Kechaou, Courtois, & Bonazzi, 2013; Chiralt et al., 2001; Giovanelli, Brambilla, Rizzolo, & Sinelli, 2012; Ojha et al., 2017; Tortoe, 2010). Most of the fruits are wasted in farm despite its high value. However, lack of proper processing techniques and unawareness of its health benefits, fruit like mulberry, rich in anthocyanins are not properly utilized. So, it is necessary to identify the proper technology which not only enhances the shelf‐life of mulberry fruit but also identify the proper usage.

One of the major problems of yoghurt industry is wheying‐off. Whey separation (syneresis) is the expulsion of whey from three‐dimensional networks, which become visible on the surface (Lucey, Munro, & Singh, 1997; Prothon et al., 2001). This results in short shelf‐life of yoghurt due to lack of body and texture. Whey separation is affected by various factors such as pH, acidity, total solid content, microbial culture, the addition of stabilizers, and hydrocolloid (Athar, Shah, & Khan, 2000; Celik & Bakirci, 2003; Koksoy & Kilic, 2004; Lee, Kim, Watkins, & Batt, 1994; Selvamuthukumaran & Farhath, 2014). Various literature cited that dried or partially dried fruit can improve the stability of yoghurt due to the low acidity and higher solid content compared with fruit juice (Athar et al., 2000; Celik & Bakirci, 2003; Sarmini, Sinniah, & Silva, 2014; Selvamuthukumaran & Farhath, 2014). It is not only necessary to prevent syneresis but also increase the health benefits of The research aimed to utilized the under‐rated mulberry fruit (in context to Nepal) as a potential source of bioactive components in yoghurt and also to minimize syneresis. So, this research was designed to study the stability and phytochemicals of Osmo‐dried mulberry incorporated yoghurt.

## 2 MATERIALS AND METHODS

### 2.1 Raw materials collection

Fresh mulberry fruits, grown in the field of Damauli, Nepal, which lies at an altitude of 650 m from sea level, were transported to the laboratory in the icebox and then refrigerated below 5°C. Standard direct vat set (DVS) freeze‐dried yoghurt culture (*S. thermophilus* and *L. bulgaricus*) was provided by Dairy Development Corporation (DDC), Nepal. Sugar was purchased from the local market of Kathmandu, Nepal, whereas standard milk (solid‐not‐fat: 10%, Fat: 3% and Protein: 3.6%) was obtained from DDC, Nepal.

### 2.2 Chemicals and reagents

2,2‐diphenyl‐1‐picrylhydrazyl (DPPH) was purchased from Sigma‐Aldrich Company, Germany. Phenol reagent was purchased from Finar Limited, India. Gallic acid was purchased from LOBA Chemie, India. Methanol was purchased from Fisher Scientific, India. Analytical grade chemicals were used for the analytical purpose. The spectrophotometer used was of model GENESYSTM 10S Vis Spectrophotometer from Thermo ScientificTM, Germany.

### 2.3 Preparation of Osmo‐air‐dried mulberry

Fruit (2.0 kg) was steeped in 0.1% potassium metabisulfite solution (1:1.5) for 10 min and then drained well with the help of muslin cloth followed by a quick rinse with potable water and treated with 2% Ca(OH)_2_ (1:1.5) for 10 min for texture improvement purpose as suggested by (Chavan & Amarowicz, 2012). Pretreated fruits were immersed in an osmotic solution of commercial sucrose (55° Bx) for about 5 hr at 40°C (Talens, Escriche, Martínez‐Navarrete, & Chiralt, 2003). The whole fruit was drained and was spread in monolayer thickness on a stainless steel sieve and dried in the convective dryer at 60 ± 5°C for 8 hr. The quality parameter and bioactive component of Osmo‐dried mulberry used were listed in Table 1.

**Table 1:** Physiochemical characteristics of mulberry fruit (Osmo‐air‐dried)

Analysis parameters	Osmo‐air‐dried fruit
Moisture content (%)	9.59 ± 0.40
Total dry weight (%)	90.40 ± 0.04
TSS (%)	29.33 ± 0.57
Vitamin C (mg%)	9.6 ± 0.26
Total phenols (mg/100 g db)	722.33 ± 3.33
Total anthocyanin (mg cyanidin‐3‐O‐glucoside equivalents (CGE)/100 g db)	154.86 ± 3.32
Antioxidant activity (% radical scavenging activity (RSA))	74.33 ± 1.53

The values in the table are the arithmetic mean of triplicate with standard deviation (±).

### 2.4 Preparation of yoghurt

Skim milk powder was added at 4% to preheated standardized milk (SNF: 10%, Fat: 3%, protein: 3.6%) at 45°C to increase total milk solid to 16.34%. The milk base was heated at 85 ± 2°C for 30 min in sterile stainless steel utensil and then cooled to 40°C. Standard DVS freeze‐dried yoghurt culture was inoculated according to the specification (78 g/2,000 L‐normal yoghurt). The cultured milk was filled to 100 ml in sterile plastic cups containing dried mulberries at the rate of 12% (from the trial) to develop set type fruit yoghurts, and control without fruit was also prepared. All these samples were then incubated at 42 ± 2°C (for about 3 hr) till the acidity (in terms of lactic acid) of yoghurt reached to about 0.9%. When yoghurt was well set they were cooled to 4°C. The samples were coded as NCY (normal control yoghurt‐without fruits) and NFY (normal fruit yoghurt).

### 2.5 Physiochemical analysis

Mulberries (Osmo‐air‐dried) were analyzed for various parameters. The moisture content, dry matter, total soluble solid (TSS), and pH were analyzed as per (AOAC, 2005). The yoghurt was analyzed for moisture content, total solids, acidity (as lactic acid), pH, and fat content, according to (AOAC, 2005). For TSS, mulberry and water (1:1) were squeezed to extract the juice and filter through cheesecloth for determination.

### 2.6 Analysis of bioactive properties (phytochemicals)

Ascorbic acid of fruits and yoghurt samples was determined according to (AOAC, 2005) using 2,6‐dichlorophenol indophenol visual titration method.

20 g of each sample was ground with 80% methanol (30 ml) and was agitated in a mechanical shaker for 20 min and then was filtered through Whatman No. 1 filter paper. Two more extraction cycle for 20 min was carried out, and volume was made 100 ml in a volumetric flask as suggested by (Kostic et al., 2013) with some modification.

The total phenol content of sample extracts was measured using the Folin–Ciocalteu method, as described by (Mahdavi, Nikniaz, Rafraf, & Jouyban, 2011). The absorbance was measured using an automated UV–VIS spectrophotometer at 750 nm. The results were expressed as mg of gallic acid equivalents (GAE) per 100 g of sample. The total anthocyanin content of the extracts was determined using the pH differential methods (Mónica Giusti & Wrolstad, 2005) using UV–VIS spectrophotometer. Results were expressed as mg of cyanidin‐3‐O‐glucoside equivalents (CGE) per 100 g. The antioxidant activity of the extract was determined by the free radical scavenging activity using DPPH assay (Mahmood, 2011; Stajcic et al., 2012). The antioxidant activity was calculated as the percent inhibition caused by the hydrogen donor activity of each sample according to the following Equation (1).

(1) $$\begin{array}{cc} \mathrm{Scavenging} & \mathrm{activity}(\%)=1-\frac{\mathrm{absorbance}\;\mathrm{of}\;\mathrm{the}\;\mathrm{sample}}{\mathrm{absorbance}\;\mathrm{of}\;\mathrm{the}\;\mathrm{blank}\times 100} \end{array}$$

### 2.7 Storage stability

The control and fruit yoghurt were taken out of refrigerated storage (4°C) on every 3 days interval and were analyzed for pH, acidity, syneresis, and viable lactic acid bacteria (LAB) count. Syneresis was measured by the method described by Amatayakul, Sherkat, and Shah (2006) with slight modification. For syneresis, approximately 15 g of yoghurt gel was weighed and drained on muslin cloth for 30 min at room temperature (25°C). The syneresis was expressed as the percentage of the whey separated from gel over initial weight of the gel.

Viable lactic acid bacteria (LAB) count was determined by the method described by Ramakant (2006). For enumeration of LAB, De man Rogosa and Sharpe agar was used, and colonies were interpreted as colony‐forming units (CFU/g of yoghurt sample).

### 2.8 Statistical analysis

The data were analyzed with one‐way analysis of variance (ANOVA) (no blocking) using GenStat programming at the 5% level of significance (*p* < .5), and *t*‐test using Microsoft Excel 2007.

## 3 RESULTS AND DISCUSSION

### 3.1 Chemical composition of final yoghurts

The chemical composition of yoghurt with and without fruit is shown in Table 2. Control yoghurt has significantly lower total solid content (16.33%) while yoghurt receiving fruits had higher total solid content (23.16%) as fruit yoghurt was formulated with dried fruits. The fat content of control samples was high (17.21%) in comparison with fruit containing samples (11.84%), and this might be due to no fat contributing fruit which caused a decrease fat content which was similar to that of Temiz, Tarakci, Karaden, and Bak (2012). The addition of fruits significantly increased the total titratable acidity of yoghurts, along with reduced pH values, and the reduction was mainly due to low pH values of fruits. The increased total soluble solid content of fruit incorporated yoghurt might be due to the addition of Osmo‐air‐dried fruit. It was clear that the addition of fruits in yoghurt increased total dry weight (TDW) (%), TSS (%), and acidity, whereas pH and fat content decreased in the final product. Obtained results were in agreement with those reported by (Tarakçi & Küçüköner, 2003).

**Table 2:** chemical composition of final yoghurt

Parameters	NCY	NFY
Moisture (%)	83.66 ± 0.57^a^	76.83 ± 0.76^b^
Total dry weight (TDW) (%)	16.33 ± 0.57^a^	23.16 ± 0.76^b^
Fat (% db)	17.21 ± 0.71^a^	11.84 ± 0.36^b^
Acidity (% as lactic acid)	0.84 ± 0.01^a^	0.87 ± 0.01^b^
pH	4.37 ± 0.01^a^	4.31 ± 0.01^b^
TSS (%)	9.5 ± 0.57^a^	17.67 ± 0.57^b^

The values in the table are the arithmetic mean of triplicate with standard deviation (±).

Values in the row bearing different superscript are significantly different at the 5% level of significance.

### 3.2 Phytochemical characteristics (bioactive components) of yoghurt

Phytochemical characteristics of yoghurt (with and without fruit) were analyzed, and the results were shown in Table 3. There was a significant increase in ascorbic acid, total phenols, anthocyanin, and antioxidant activity of yoghurt incorporated with fruit. Ascorbic acid was found to increase by more than 7.5 times (0.77 mg/100 g to 5.96 mg/100 g) in mulberry fortified yoghurt. Selvamuthukumaran and Farhath (2014) reported an ascorbic acid content of 20 mg/100 g yoghurt in yoghurt produce with the addition of sea buckthorn. The yoghurt formulated with mulberry fruits contained higher TPC (68.03 mg GAE/100 g) compared with control (6.63 mg GAE/100 g), which was more than 10 times. The detected TPC value of control yoghurt was in agreement with those who reported TPC 3–6 mg/100 g (Bakr, Mohamed, Tammam, & El‐gazzar, 2015) and 5.13–7.24 mg/100 g (Chouchouli et al., 2013). The TPC content in plain yoghurt might be due to break down of a phenolic side chain of milk proteins (Damin, Alcântara, Nunesb, & Oliveiraa, 2009; Shah, 2000). The clear increment of TPC in fruit yoghurt over the values of control yoghurt indicates the presence of mulberry polyphenols (phenolics, anthocyanins, and flavonoids) in the final products. A similar result was reported by Samh, Sherin, and Essam (2013). The mulberry fruit fortified yoghurt posses 7.9 mg CGE/100 g, whereas anthocyanin was not detected in normal yoghurt. Anthocyanin in fruit yoghurt was contributed by the fruit itself. Scibisz, Ziarno, Mitek, and Zare (2012) also found 12 mg/100 g of anthocyanin in yoghurt enriched with 20% blueberry preserve. Various researcher has illustrated anticancer activity, prevention of cardiovascular disease, antidiabetic role, anti‐obesity, and improved visual health from plant anthocyanins (Jayaprakasam, Vareed, Olson, & Nair, 2005; Miyake et al., 2012; Rechner & Kroner, 2005; Tsuda, Horio, Uchida, Aoki, & Osawa, 2003; Wang et al., 2009). 12–36 mg daily ingestion of anthocyanin extract has proven to improve night vision (Levy & Glovinsky, 1998). The result indicated that radical scavenging activity (RSA)% of yoghurt formulated with mulberry was highest (47.6 ± 2.51%) when compared to control yoghurt (20.73 ± 1.41%). The high antioxidant activity in fruit yoghurt than control yoghurt was most likely contributed by individual fruit phytochemical content (phenols, flavonoids, anthocyanins, and ascorbic acid) and as a result of microbial metabolic activities (Thompson, Lopetcharat, & Drake, 2007). The antioxidant activity of plain yoghurt was found to be in the range of 19% to 28.49% by different authors (Chouchouli et al., 2013; Shori & Baba, 2014). The high antioxidant activity of fruit yoghurt is a desirable characteristic that may enhance the therapeutic value of yoghurt and is reported to decrease the risk of some diseases such as cardiovascular and cancer (Kris‐etherton, Harris, & Appel, 2002).

**Table 3:** Phytochemical characteristics of yoghurt (with and without fruits)

Parameters	NCY	NFY
Ascorbic acid	0.77 ± 0.01^a^	5.96 ± 0.20^b^
Total phenols (mg GAE/100 g)	6.63 ± 0.85^a^	68.03 ± 2.86^b^
Anthocyanin (mg CGE/100 g)	–	7.9 ± 0.86
Antioxidant activity of (% RSA)	20.73 ± 1.41^a^	47.6 ± 2.51^b^

The values in the table are the arithmetic mean of triplicate with standard deviation (±).

Values in the row bearing different superscript are significantly different at the 5% level of significance.

### 3.3 Study of storage of final products on various parameters

#### 3.3.1 Change in pH and acidity

**Figure 1:**
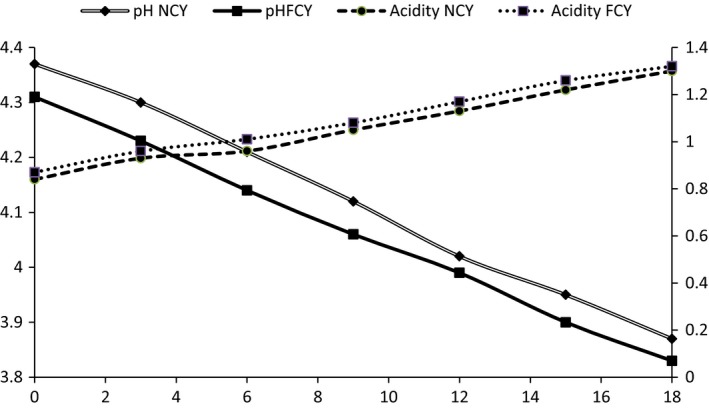
Change in acidity and pH of different yoghurt samples at refrigerated condition

The change in acidity of NCY and NFY is shown in Figure 1. The initial mean pH value and mean acidity of the yoghurt (without mulberry) were lower than those of yoghurts with fruits as the fruits tend to show little higher acid and a lower pH value. The acidity of normal yoghurt increased from 0.84% to 1.3% during 18 days of storage at refrigerated temperature, whereas pH decreased from 4.37 to 3.87. Similarly, the acidity of normal yoghurt increased from 0.87% to 1.32% during 18 days of storage at refrigerated temperature, whereas pH decreased from 4.31 to 3.85. A similar trend of a rise in acidity and decreasing pattern of pH in yoghurt with storage period and the addition of fruits were revealed by various authors (Farahat & El‐batawy, 2013; Selvamuthukumaran & Farhath, 2014). The increase in acidity of fruit yoghurt is normal phenomenon due to low pH of fruits, and this low pH causes increase in acidity (Öztürk & Öner, 1999). According to Shah and Jelen (1990), postacidification of yoghurt during storage at 4°C occurs because lactic acid bacteria are active at this temperature and produce lactic acid resulting in noticeable decreased in pH and increased acidity.

#### 3.3.2 Change in syneresis

**Figure 2:**
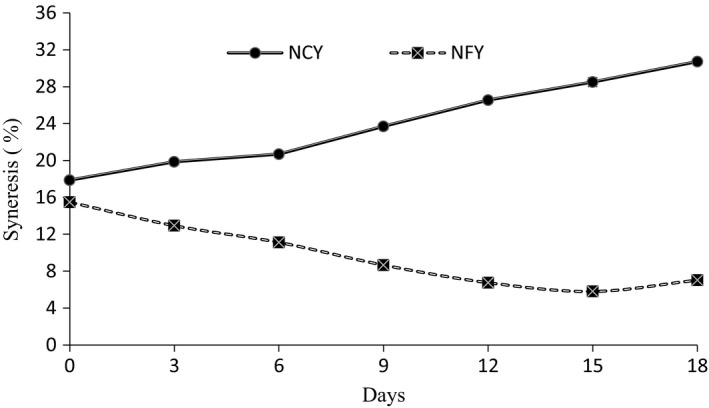
Change in syneresis of different yoghurts upon refrigerated storage

The syneresis of control and fruit yoghurt is shown in Figure 2, and the trendline is shown in Table 4. The syneresis of plain yoghurt increased from 17.56% to 30.2% in 18 days of storage while the syneresis of fruit yoghurt decreased from 15.48% to 5.8% in 15 days of storage while again increased to 7.04% in further 3 days of storage. Syneresis of normal yoghurt follows linear trendline, whereas, for fruit yoghurt, syneresis follows logarithmic trendline. However, for normal yoghurt, syneresis increased with time, whereas in the case of fruit yoghurt, negative slope indicates a decrease in syneresis with time. Izadi, Nasirpour, Garoosi, and Tamjidi (2015) also reported a decrease in syneresis of phytosterol‐enriched yoghurt with storage period.

**Table 4:** Trendline of syneresis of yoghurt with and without fruit

Parameters	NCY	NFY
Equation	Y = 2.2046X + 15.16	Y = −5.123ln(X) + 15.921
Slope	2.2046	−5.123
*R* ^2^	0.9874	0.9471

Athar et al. (2000) reported that decrease in pH value accelerates the syneresis in yoghurt. Increased syneresis in control yoghurt may be due to continued increases in acidity and decrease in pH of the product (Selvamuthukumaran & Farhath, 2014), while, yoghurts with dried fruits showed lower syneresis when compared to control yoghurt which can be correlated with absorption of unbound and free water by dried fruits. A similar result was reported by El‐Kholy, Osman, Gouda, and Ghareeb (2011) and Vahedi, Tehrani, and Shahidi (2008). Hence, this showed that dried fruits can serve as a potential source to control syneresis.

#### 3.3.3 Change in viable count of lactic acid bacteria in yoghurt

**Figure 3:**
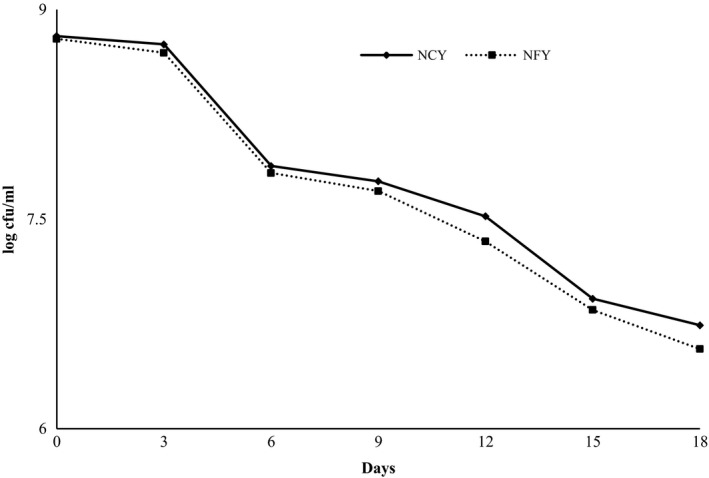
Change in viable count of lactic acid bacteria (LAB) at refrigerated storage

The pattern of change in viability in for both yoghurt (with and without fruit) stored in refrigerated condition (4°C) is shown in Figure 3. For fruit yoghurt, LAB count decreased from 8.81 to 6.74 log cfu/ml, whereas for normal yoghurt, LAB count decreased from 8.81 to 6.57 log cfu/ml. The decrease in the LAB count for both yoghurts follows a similar trend. Various authors have reported decreased in the lactic count during storage of yoghurt (Mani‐López, Palou, & López‐Malo, 2014; Panesar & Shinde, 2012). The loss of cell viability may be due to a decrease in pH during storage and accumulation of organic acid as a result of growth and fermentation (Kailasapathy, Harmstorf, & Phillips, 2008).

## 4 CONCLUSION

Osmo‐dried mulberry fruit incorporated yoghurt showed significant improvement in bioactive properties compared with normal yoghurts. These bioactive components may contribute to various health benefits. Antioxidant activity was also high, which was believed to reduce cardiovascular disease. Beside this, mulberry incorporated yoghurt also showed reduced syneresis with the time periods while in normal yoghurt, it increased with time

## CONFLICT OF INTEREST

None declared.
